# Determination of dengue high-risk areas in the Philippines: a kernel density estimation, inverse distance weighting, and ecological niche modeling

**DOI:** 10.1186/s13071-025-07200-4

**Published:** 2025-12-23

**Authors:** Kenny Oriel A. Olana, Aksara Thongprachum, Napaphat Poprom, Wengui Li, Veerasak Punyapornwithaya

**Affiliations:** 1 Doctor of Public Health Program, Faculty of Public Health, Chiang Mai University, Chiang Mai, Thailand; 2 Department of Veterinary Paraclinical Sciences, Faculty of Veterinary Medicine, Visayas State University, Baybay City, Leyte Philippines; 3 Faculty of Public Health, Chiang Mai University, Chiang Mai, Thailand; 4 College of Veterinary Medicine, Yunnan Agricultural University, Kunming, China; 5 Research Center for Veterinary Biosciences and Veterinary Public Health, Faculty of Veterinary Medicine, Chiang Mai University, Chiang Mai, Thailand; 6 Veterinary Public Health and Food Safety Centre for Asia Pacific (VPHCAP), Faculty of Veterinary Medicine, Chiang Mai University, Chiang Mai, Thailand

**Keywords:** Dengue, Ecologic niche model, Maxent, Infectious disease, Kernel density, Philippines

## Abstract

**Background:**

Dengue is an acute infectious tropical disease that poses a significant public health burden in the Philippines; however, studies employing spatial distribution modeling and ecological approaches to analyze dengue occurrence data remain limited. This study aims to determine the high-risk areas suitable for dengue occurrence and its determinants in the Philippines.

**Methods:**

Dengue case data from 2017 to 2024 were analyzed using kernel density estimation (KDE) and inverse distance weighting (IDW) spatial interpolation to characterize spatial intensity and estimate incidence in unsampled areas. An ecological niche model was developed using maximum entropy modeling, implemented through the MaxEnt software, with climatic, environmental, and socioeconomic predictors. Model performance was evaluated using the area under the curve (AUC), and predictor importance was assessed using jackknife testing.

**Results:**

Results show highest intensity in 2019 and consistent high case density in the National Capital Region (NCR). Meanwhile, high predicted incidence rates were consistently exhibited in northern Luzon. The maximum entropy model had a strong performance in predicting the suitable areas for dengue with a mean area under curve (AUC) of 0.847. Nighttime lights (32.3%), land cover (31.1%), and population density (9.4%) significantly contributed to the model. The NCR was found to be a high-risk suitable area for dengue occurrence along with some parts of other provinces.

**Conclusions:**

This study represents the first application of ecological niche modeling to dengue in the Philippines. The integration of KDE, IDW, and maximum entropy model provides a robust framework for identifying high-risk areas and key determinants, emphasizing the role of urbanization in dengue distribution. These findings are valuable to authorities for an informed risk-based surveillance, genotype-specific monitoring, and decision-making for geospatially targeted disease risk management.

**Graphical Abstract:**

**Figure:**
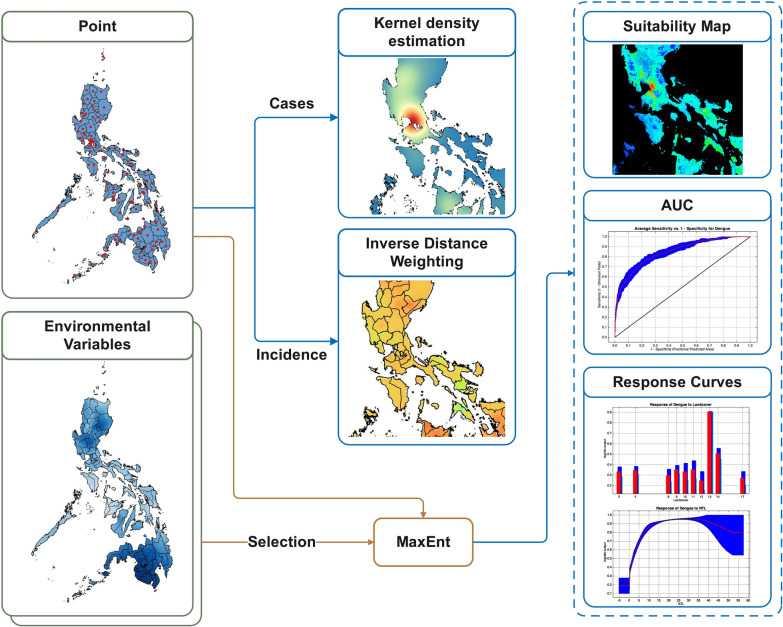

**Supplementary Information:**

The online version contains supplementary material available at 10.1186/s13071-025-07200-4.

## Background

In the past three decades, the global burden of dengue has grown dramatically, making it the fastest-growing mosquito-borne viral disease [1]. Since 1990, the incidence of dengue has more than doubled from 26.45 million cases to global annual estimates ranging from 58 to 104 million cases [1, 2]. Notably, some models suggest that the total number of infections including asymptomatic cases may reach as high as 390 million per year [3]. Mortality and disability-adjusted life years (DALYs) have also risen, with deaths increasing from around 14,000 in 1990 to over 29,000 in 2021 [4, 5]. Along with other mosquito-borne diseases, dengue threatens more than 5.2 billion people across 132 countries and territories [6, 7]. According to the World Health Organization (WHO) [8], over 14.6 million cases and more than 12,000 dengue-related deaths were reported in 2024. From January to July 2025, more than 4 million dengue cases and over 3000 associated deaths have been reported from 97 countries. The United Nations Sustainable Development Goals (SDGs), aimed to halt epidemics of dengue and other neglected tropical diseases by 2030; however, the world is still not on track to achieve such goals, as the incidence of dengue has continued to increase in recent years [9, 10].

Dengue is an acute tropical disease caused by dengue virus (DENV) under the family *Flaviviridae* [6, 11]. Predominantly transmitted by *Aedes aegypti* mosquito and secondarily by *Aedes albopictus* [10, 12], the disease continuously spreads geographically as the vectors expand their geographic range owing to various factors, including climate change, human mobility, and urbanization [13–15]. In endemic countries, socioeconomic factors such as inadequate water management and ineffective waste management systems exacerbate the spread of dengue [16]. South Asia, Southeast Asia, and tropical Latin America are the most severely affected regions [1]. Considered now as endemic in over 100 countries, dengue poses a major global health threat.

The Philippines is among the countries most vulnerable to dengue epidemics. The first case of dengue hemorrhagic fever (DHF) was reported in the Philippines in 1953 and it further spread to countries in Southeast Asia and the Western Pacific region [11]. Since then, dengue has imposed a significant health and economic burden on the country [17]. In 2019, 437,089 cases and 1689 deaths from dengue were recorded—the highest case count and mortality rate in Asia [18, 19]. In recent years, the Philippines has reported the highest number of dengue cases among Association of Southeast Asian Nations (ASEAN) countries, with 195,603 cases in 2023 and 413,960 cases in 2024, respectively [20]. However, the true burden is far greater; models suggest that the adjusted average number of dengue cases in the Philippines yearly was estimated to be 842,867 of which 53% of the reported cases were diagnosed as DHF or dengue shock syndrome (DSS) and 47% were diagnosed as dengue fever (DF) [17]. A great proportion of this burden comes from children aged 5–14 years [21]. Previous studies have demonstrated a strong correlation between rainfall levels and increased dengue incidence in the country [22]. Seasonal epidemics typically peak from July to September, coinciding with the rainy season. The main vector responsible for dengue transmission in the Philippines is *Aedes aegypti*, which is predominant in urban areas, and *Aedes albopictus* as a secondary rural vector [23, 24]. All four serotypes of DENV (1–4) are present in the Philippines [25]. DENV-2 is considered the cosmopolitan genotype in the Philippines as it continues to circulate in metropolitan areas [26]. Geographically, the island of Luzon is the major source of DENV dispersal, while Mindanao is the hub for DENV-4. Interestingly, the predominant dengue serotype has varied by year, with DENV-1 being most common in 2016, DENV-3 in 2017, and DENV-2 in 2021 [18, 27]. A shift in DENV-4 genotype has been reported, suggesting the role of genetic evolution in sustained transmission [28]. Currently, the Philippines represents a substantial proportion of the dengue burden in Southeast Asia, which constitutes 75% of the global disease burden [29, 30]. Therefore, its dengue control and prevention strategies must be substantially strengthened.

Spatial epidemiological tools are becoming more important to model infectious diseases. Kernel density estimation (KDE), inverse distance weighting (IDW) spatial interpolation, and ecological niche modeling (ENM) are useful methods to explore and model disease high risk areas [31, 32]. KDE is an extensively used nonparametric method that estimates the underlying density of spatial data by placing a smooth kernel function at each data point and summing the contributions across the study area, resulting in a continuous surface that highlights areas of higher or lower density [31, 33, 34]. It helps to identify patterns and outbreak clusters and enables accurate prediction of disease incidence and spatial risk assessment [31]. Recent applications of KDE were observed in various health events, such as dengue [35, 36], chikungunya [37], and coronavirus disease 2019 (COVID-19) [38], among others. IDW is a standard geostatistical tool that interpolates disease risk at unsampled locations on the basis of proximity to known cases, assuming closer points have more influence. The method is commonly used in epidemiology to create continuous risk maps from discrete case data, supporting resource allocation and surveillance.

ENM, however, is a key ecological approach used to map disease transmission [39]. It predicts a species’ fundamental niche on the basis of a set of environmental predictor variables and projects this niche onto geographic space [40]. Furthermore, it is widely used for potential disease spread, and generating disease risk maps, especially ENM governed by maximum entropy [32, 39, 40]. Maximum entropy modeling is a common technique of ENM that allows for accurate predictions using presence-only data and environmental variables. Fundamentally, it examines relationships between environmental variables and disease occurrence to understand disease biology, while also predicting potential geographic distribution with explicit consideration of data uncertainty [39, 41]. Maximum entropy exhibited superior performance over other methods and was used to predict emerging infectious diseases [32, 40]. To date, no studies in the Philippines have applied ecological niche models to predict emerging infectious diseases [32].

Despite the Philippines being a hyperendemic country, the applications of advanced models to understand the spatial epidemiology of dengue remains limited. Therefore, the study aimed to delineate high-risk areas for dengue occurrence, assess temporal and spatial distribution patterns, and identify the most influential determinants of dengue suitability in the Philippines using KDE, IDW, and maximum entropy model. Comprehensive integration of these methods provides a powerful framework for identifying high-risk areas and their determinants, which is essential to support targeted surveillance and genotype monitoring and inform risk-based interventions. This study demonstrates the application of integrated spatial models in a dengue hyperendemic country.

## Methods

### Study area

The Philippines is an archipelago in Southeast Asia located in the Western Pacific Ocean. The country consists of 7641 islands that are classified into three main island groups: Luzon, Visayas, and Mindanao [42]. Its climate is tropical with wet and dry seasons driven by monsoons, which occur from December to April (Northeast) and May to November (Southwest) [43]. However, Corporal-Lodangco and Leslie [44] reported that the climate zones of the country is more complex. Being near the equator, its climate is characterized by relatively high temperatures, high humidity, and abundant rainfall [45]. The Philippines has a mean annual temperature of 26.6 °C, average monthly relative humidity ranging from 71% in March to 85% in September, and annual rainfall between 965 and 4064 mm. With a population exceeding 115 million, its national estimated population density is 389 persons per square kilometer [46]. Urbanization is accelerating in the country owing to rapid socioeconomic development [47].

### Occurrence data

Considering the wide distribution of dengue in the Philippines and the scarce information on the exact location of vectors that are presumed to be widespread across the country [48], a national epidemiological dataset from 2017 to 2024 consisting of the number of reported dengue cases was obtained from the Philippine Department of Health, Epidemiology Bureau. Underreporting or surveillance biases may occur at various organizational levels. However, significant improvements in the reporting sensitivity and completeness were noted since the implementation of the Philippine Integrated Disease Surveillance and Response Information System (PIDSR-IS) in 2007 [49]. The location of provinces and cities where dengue cases have been reported was derived and used in the study. The incidence per 100,000 people of each province was generated yearly by dividing the annual total reported dengue cases by the median estimated population of the respective year and multiplying by 100,000. Centroid coordinates were then generated using QGIS version 3.36 Maidenhead [50] for each location (Additional file 1: Supplementary Table S1). A total of 121 locations with dengue presence across the country were identified and used for ecological niche modeling (Additional file 2: Supplementary Fig. S1).

### Kernel density estimation (KDE)

To determine dengue high-risk areas based on the number of cases, KDE was performed. KDE applies a bivariate probability density function to the point data to produce estimates of the intensity of a spatial point process [51, 52]. The method relies on the assumption that events closer in space have a greater influence on the density estimate at a specific location, thereby enabling localized smoothing on the basis of spatial proximity. KDE does not assume any specific spatial distribution, making it flexible for various datasets [53, 54]. The resulting heat maps depict warmer zones as areas with a higher density of cases.

To perform the analysis, the density() function of the “ks” package in R software (R Core Team, Vienna, Austria) was employed. Centroid coordinates of reporting provinces and cities were utilized as points and the number of cases as weights. Among the KDE parameters, the bandwidth is the most critical criterion for identifying the most suitable density surface [54, 55]. Considering this, the optimal bandwidth for each year was calculated using the profile pseudolikelihood method via the bw.ppl() function of the “spatstat” package in R software (R Core Team, Vienna, Austria) rather than relying on default settings or visual evaluation. This method determines the value of bandwidth that maximizes a pseudolikelihood function derived from the observed spatial distribution, which balances the smoothness (oversmoothing versus undersmoothing) of density surface. Raster products from R were exported to QGIS version 3.36 Maidenhead [50] to generate the yearly KDE heat maps from 2017 to 2024. Detailed results of bandwidth selection are provided in Additional file 1: Supplementary Table S2, Additional file 3: Supplementary Fig. S2, and Additional file 4: Supplementary Fig. S3.

### Inverse distance weighting (IDW) spatial interpolation

To generate risk maps based on incidence and interpolate for unsampled areas, IDW spatial interpolation was performed. IDW is a deterministic spatial interpolation model that is based on the assumption that the attribute value of an unsampled point is the weighted average of known values within the neighborhood, and the weights are inversely related to the distances between the prediction location and the sampled locations [52, 56]. IDW is straightforward, easy to interpret, and useful for creating risk maps when data are irregularly spaced [56, 57]. The weighting coefficient is a key parameter; as it increases, distant points have less influence on the interpolated value, causing estimates to more closely reflect the nearest observed point.

To perform IDW, data of incidence and centroid coordinates in CSV files were imported in QGIS version 3.36 Maidenhead [50] as point layer. Incidence per 100,000 population was log transformed using base 10 with the field calculator and generated as a new column. This was done to ensure comparability across different years. The IDW function in QGIS processing toolbox was then utilized to generate the spatial interpolation models using the logged incidence as interpolation attribute. These models were presented as risk maps yearly from 2017 to 2024, with warmer zones characterized by higher logged incidence.

### Ecologic niche model

An ecologic niche model was developed to further delineate the extent of risk areas with consideration of climatic, environmental, and topographic variables. This model predicts the high-risk or suitable areas for dengue occurrence and determines the factors associated with it.

#### Variable collection

Climatic, environmental, and topographic variables affect habitat conditions for *Aedes sp.* and other mosquito vectors’ survival [58–63] and suitable habitat for mosquito-borne viruses [64, 65]. These variables have been widely used in ecological niche modeling and were also used to model dengue occurrences [66–69]. Socioeconomic or anthropogenic variables were also explored in the study, as it provides a more comprehensive approach to niche modeling [16, 63, 67, 70, 71]. Hence, we draw our model from these variables.

In the study, we selected 30 variables, including 19 bioclimatic, 9 environmental, and 2 socioeconomic variables, that could have an influence on the distribution of dengue throughout the archipelago considering biological plausibility (Table 1). Google Earth Engine (GEE), a web-based interactive development environment with a JavaScript API that provides access to extensive satellite imagery and data, was utilized to collect the variables [72]. In the GEE platform, the study area was first delineated, and a value per pixel using an algorithm (e.g., minimum, maximum, mean, or sum) of each variable between 2017 and 2024, except for bioclimatic variables which encompass climate data from 1970 to 2000, was computed. All the variables were exported in CSV and TIF formats with 1000-m resolution. Correlation analysis was then conducted among the 19 bioclimatic variables using the basic functions of R software (R Core Team, Vienna, Austria) (Additional file 5: Supplementary Fig. S4). Variables with a Pearson correlation coefficient > 0.70 and a variance inflation factor (VIF) value > 8 were excluded from the model development [73]. Finally, 13 remaining variables were identified and used for ecologic niche modeling, which are bio02, bio04, bio13, bio18, bio19, digital elevation model, land cover, normalized difference water index, nighttime lights, maximum Palmer drought severity index, population density, maximum vapor pressure deficit, and maximum wind speed. These variables were selected to capture key climatic, environmental, and human factors known to influence dengue transmission dynamics. They collectively provide insights into mosquito habitat suitability, breeding site availability, human exposure risk, and the climatic conditions that drive or inhibit outbreaks. These variables were then converted to ASCII files using QGIS version 3.36 Maidenhead [50].

**Table 1:** Summary of variables and data sources in the study

	Variable name		Dataset name
Climatic			
Bio1	Annual mean temperature		WORLDCLIM/V1/BIO
Bio2	Mean diurnal range (mean of monthly [max temp—min temp])		WORLDCLIM/V1/BIO
Bio3	Isothermality (BIO2/BIO7) (× 100)		WORLDCLIM/V1/BIO
Bio4	Temperature seasonality (standard deviation × 100)		WORLDCLIM/V1/BIO
Bio5	Maximum temperature of warmest month		WORLDCLIM/V1/BIO
Bio6	Minimum temperature of coldest month		WORLDCLIM/V1/BIO
Bio7	Temperature annual range (BIO5–BIO6)		WORLDCLIM/V1/BIO
Bio8	Mean temperature of wettest quarter		WORLDCLIM/V1/BIO
Bio9	Mean temperature of driest quarter		WORLDCLIM/V1/BIO
Bio10	Mean temperature of warmest quarter		WORLDCLIM/V1/BIO
Bio11	Mean temperature of coldest quarter		WORLDCLIM/V1/BIO
Bio12	Annual precipitation		WORLDCLIM/V1/BIO
Bio13	Precipitation in wettest month		WORLDCLIM/V1/BIO
Bio14	Precipitation in driest month		WORLDCLIM/V1/BIO
Bio15	Precipitation seasonality (coefficient of variation)		WORLDCLIM/V1/BIO
Bio16	Precipitation in wettest quarter		WORLDCLIM/V1/BIO
Bio17	Precipitation in driest quarter		WORLDCLIM/V1/BIO
Bio18	Precipitation in warmest quarter		WORLDCLIM/V1/BIO
Bio19	Precipitation in coldest quarter		WORLDCLIM/V1/BIO
Environmental			
DEM	Digital elevation model	projects/sat-io/open-datasets/FABDEM	
Landcover	Land cover	MODIS/006/MCD12Q1	
LST	Land surface temperature	MODIS/006/MOD11A2	
NDVI	Normalized difference vegetation index	MODIS/006/MOD13A2	
NDWI	Normalized difference water index	COPERNICUS/S2	
PDSI	Palmer drought severity index	IDAHO_EPSCOR/TERRACLIMATE	
Slope	Slope	projects/sat-io/open-datasets/FABDEM	
Vaporpressuredeficit	Vapor pressure deficit	IDAHO_EPSCOR/TERRACLIMATE	
Windspeed	Max wind speed	IDAHO_EPSCOR/TERRACLIMATE	
Socioeconomic			
NTL	Nighttime lights	NOAA/VIIRS/DNB/-MONTHLY_V1/VCMSLCFG	
PopulationDensity	Population density	WorldPop/GP/100 m/pop	

*MODIS* Moderate Resolution Imaging Spectroradiometer, *NOAA* National Oceanic and Atmospheric Administration, *VIIRS* Visible Infrared Imaging Radiometer Suite

#### Ecological niche modeling

The maximum entropy modeling approach was employed to predict the current distribution of dengue occurrences in the study. This approach is a machine learning tool that utilizes presence-only data to predict distributions governed by the principle of maximum entropy. Maximum entropy model estimates the potential distribution of a species by determining the distribution of the maximum entropy (i.e., closest to uniform), with constraints imposed by the observed spatial distributions of the species and the environmental conditions [74]. The model is mathematically expressed as [75, 76]:

$${P}_{\omega }(y\mid x)=\frac{1}{{Z}_{\omega }(x)}\mathrm{exp}\left(\sum_{i=1}^{n} \omega ifi(x\mid y)\right)$$

$${Z}_{\omega }(x)=\sum_{y} \mathrm{exp}\left(\sum_{i=1}^{n} \omega ifi(x\mid y)\right)$$

where ${P}_{\omega }(y\mid x)$ is the conditional probability of output $y$ given input $x$; $x$ represents the environmental variable input into the model, which is parameterized by weights $\omega$; $y$ represents the predicted geographical area; $fi(x\mid y)$ represents feature functions that extract specific characteristics or patterns between $x$ and $y$; $\omega i$ are the weights for each feature function $fi(x\mid y)$; and ${Z}_{\omega }(x)$ is the normalization constant that ensures the probabilities sum to 1 over all possible outputs $y$.

The modeling was performed via standalone MaxEnt software version 3.4.4 (https://biodiversityinformatics.amnh.org/open_source/maxent/); 80% of the occurrence record was used for training the model, while the remaining 20% was used to evaluate the model to ensure robust performance assessment. The logistic output format was chosen for its interpretability, and feature selection was set to automatic to allow the model to adjust complexity on the basis of data characteristics. The regularization multiplier of one was set to minimize overfitting, and 10,000 background points were used to adequately sample environmental conditions. To address model uncertainty and ensure reproducibility, the run type used was bootstrap and the random seed was ticked. To prevent duplication and spatial autocorrelation in the data, the “remove duplicate presence records” function in the MaxEnt software was ticked in the analysis to ensure that there was only one record in a grid [77]. The model was run in ten replicates providing stable estimates of predictive performance, and the average was taken as the final prediction. The area under the receiver operating characteristic curve (AUC) criteria were used to assess model performance, with AUC values ranging from 0 to 1. Better performance of the model is indicated by a higher AUC. An AUC greater than 0.5 indicates a superior predictive capacity of the model than a model of random prediction [74].

## Results

### Kernel density estimation

**Fig. 1:**
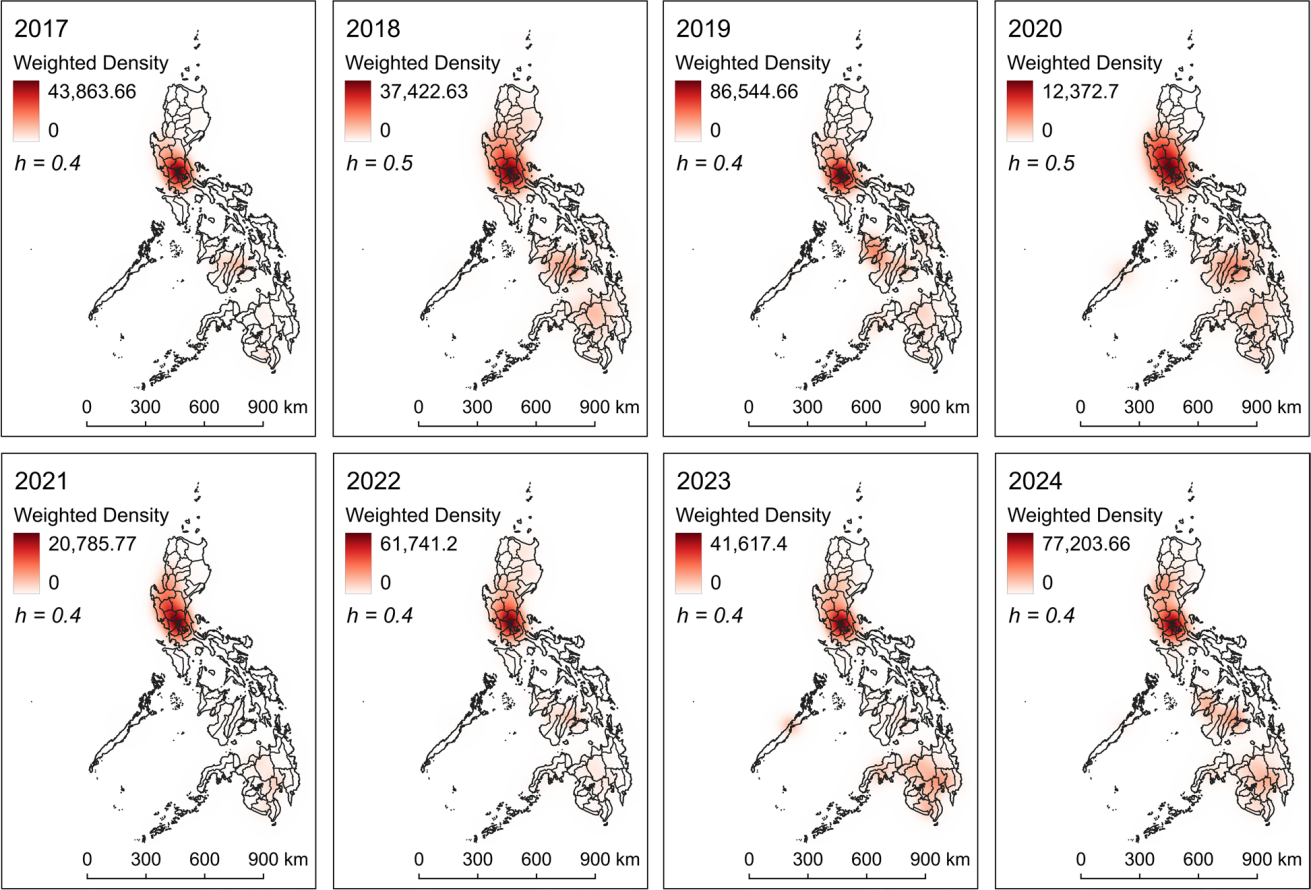
Case weighted kernel density estimates of dengue cases in the Philippines from 2017 to 2024. *h* represents the optimum bandwidth used for a specific year. The subnational administrative boundaries of the Philippines were obtained from the United Nations Office for the Coordination of Humanitarian Affairs [available at: https://data.humdata.org/dataset/cod-ab-phl?]

**Fig. 2:**
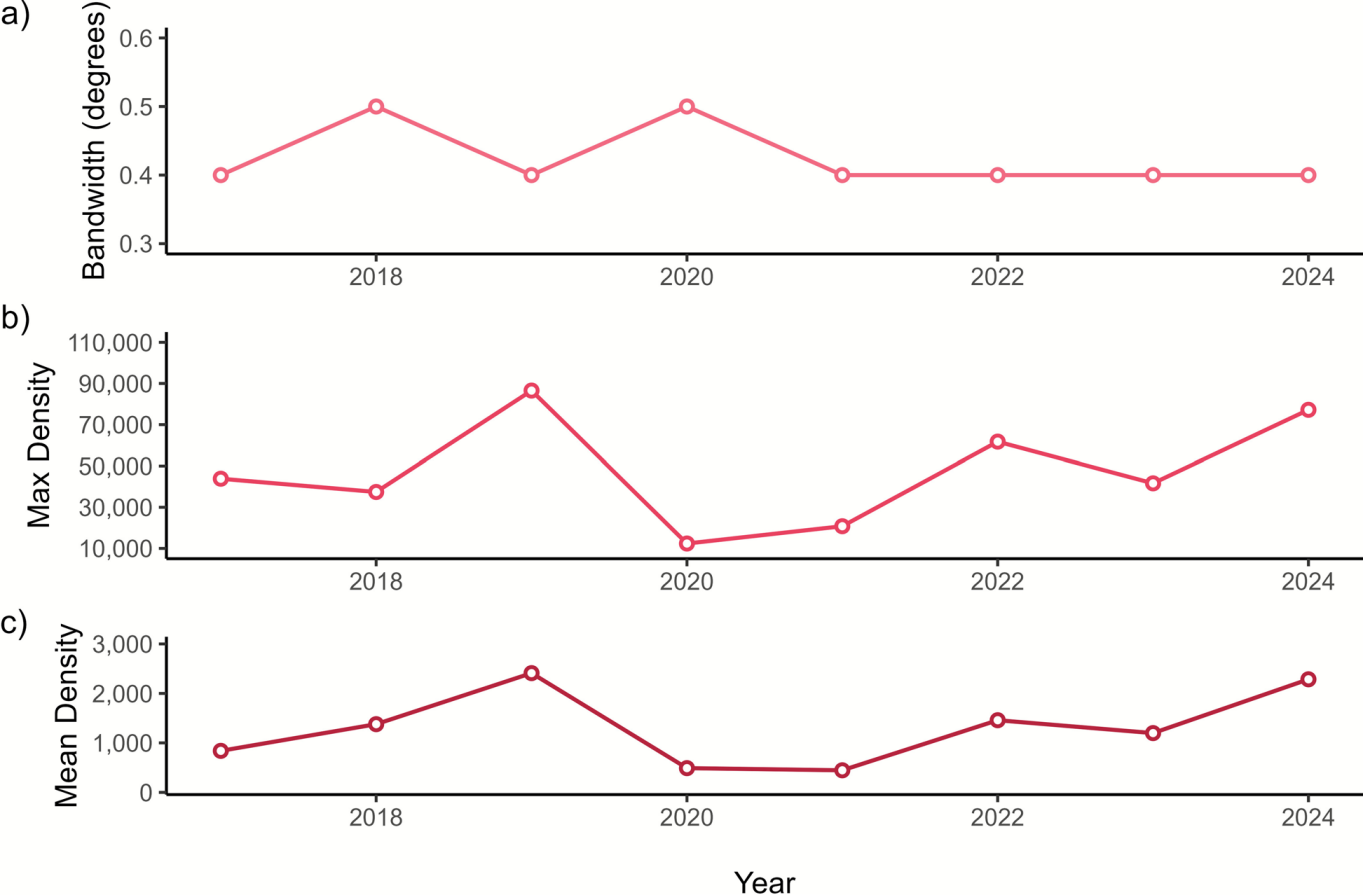
Temporal analysis of kernel density estimates of dengue cases in the Philippines from 2017 to 2024. **a** optimal bandwidth by year, **b** maximum density by year, and **c** mean density by year

KDE results show the intensity of dengue cases in the Philippines. Heat maps of dengue cases from 2017 to 2024 show consistently high weighted density in the NCR (Fig. 1, Additional file 4: Supplementary Fig. S3). Apparently, the overall intensity and mean density was the highest in 2019, indicating the national epidemic that occurred during the year [18] (Fig. 2, Additional file 1: Supplementary Table S2). Low mean density was observed in 2020 and 2021.

### Inverse distance weighting (IDW) spatial interpolation

**Fig. 3:**
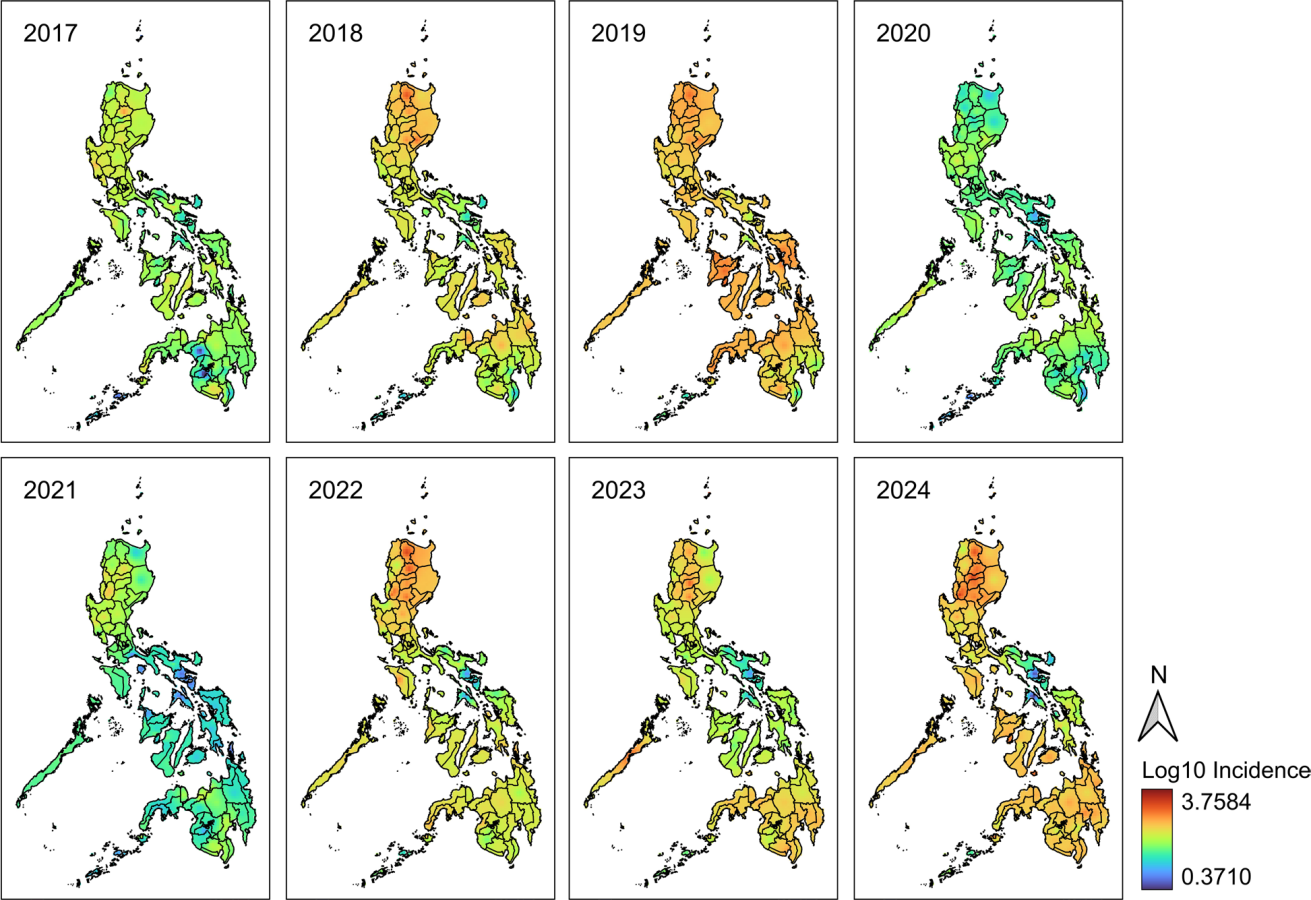
Inverse distance weight (IDW) linear interpolation of dengue incidence from 2017 to 2024. The subnational administrative boundaries of the Philippines were obtained from the United Nations Office for the Coordination of Humanitarian Affairs [available at: https://data.humdata.org/dataset/cod-ab-phl?]

Results of IDW spatial interpolation show the estimated risk or incidence in the Philippines from 2017 to 2024. The analysis revealed year-to-year differences reflecting varying transmission intensity across the years (Fig. 3). In 2019, a vast portion of the country had high predicted incidence values. A similar intensity was observed in 2024, whereas smaller areas of the country exhibited high intensity in 2018, 2022, and 2023. During these years, high incidence is consistently observed in northern Luzon.

### Maximum entropy model

**Fig. 4:**
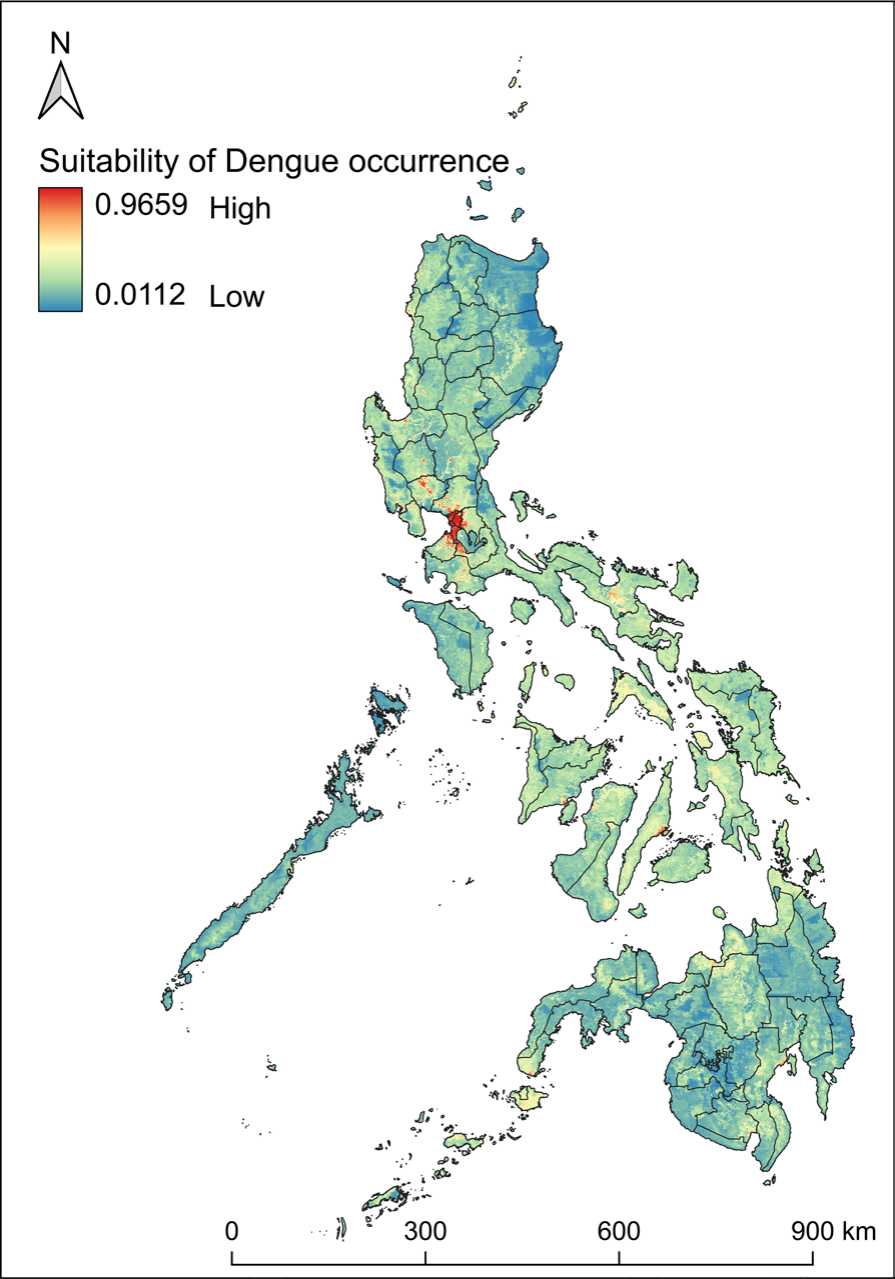
Suitability map for dengue in the Philippines. The warmer colors depict areas of high suitability, while cooler colors depict areas of low suitability

The maximum entropy model performed well having a mean AUC of 0.847 ± 0.0596, with a mean training AUC of 0.847 ± 0.0243 and a test AUC of 0.730 ± 0.0389, which are consistently above the random prediction indicating robust prediction of dengue distribution suitable areas (Additional file 6: Supplementary Fig. S5). Three variables were considered important in the spatial spread of dengue, which are nighttime lights (32.3%), land cover (31.1%), and population density (9.4%) (Table 2). Response curves of each variable show the relationships between the important factors and the suitability of dengue occurrence (Additional file 7: Supplementary Fig. S6). A nighttime light logged value ranging from 8 to 40 average radiance (Supplementary Fig. S6a), a type 13 land cover (urban or built-up areas) (Supplementary Fig. S6b), and a population density value from 1 to 7 people per square kilometer define highly suitable areas for dengue occurrence in the Philippines (Supplementary Fig. S6c). The jackknife test further confirms the influence of these variables in the probability of dengue (Additional file 8: Supplementary Fig. S7). The maximum entropy model for dengue occurrence is shown in Fig. 4. The high-risk areas of dengue were concentrated in the NCR, which extends to the provinces of Rizal, Bulacan, and Pampanga to the north and the provinces of Cavite, Laguna, and Batangas to the south. Other highly suitable areas for the prevalence of dengue in the country were found in some parts of Batanes, Ilocos Norte, Ilocos Sur, La Union, Benguet, Pangasinan, Nueva Ecija, Tarlac, Zambales, Bataan, Camarines Sur, Albay, Iloilo, Negros Occidental, Cebu, Misamis Oriental, Davao del Sur, and Zamboanga Sibugay.

**Table 2:** Relative rates of variable contribution

Variable	Variable contribution (%)
NTL	32.3
Land cover	31.1
Population density	9.4
Bio4	3.9
DEM	3.5
Bio2	3.0
Maximum wind speed	2.9
Bio19	2.8
Maximum PDSI	2.6
Bio18	2.6
Maximum vapor pressure deficit	2.0
NDWI	1.9
Bio13	1.8

## Discussion

The Philippines is a dengue-hyperendemic country; however, such status warrants the investigation of high-risk areas and potential determinants. This study aimed to identify the high-risk, suitable areas for dengue occurrence in the Philippines and its underlying determinants using ecological niche modeling. Year-to-year differences in the number of dengue cases were found in the study with high density invariably found in the NCR. Predicted incidence values, however, were generally higher in the northern Luzon. The maximum entropy model had a strong performance in predicting the suitable regions and identified NCR as the region to have the highest suitability for dengue prevalence with nighttime lights, land cover, and population density as the most influential factors. These findings underscore that socioeconomic factors indicating urbanization are significantly associated with the risk and probable spread of dengue. The high-risk areas identified have significant probability of dengue outbreaks and are important for targeted intervention and virologic surveillance. Furthermore, the importance of planning and readiness for the occurrence of dengue outbreaks in highly urbanized and densely populated areas is strongly emphasized.

Urbanization and the growing population plays a crucial role in dengue spread in the Philippines [21]. The increasing urbanization in Philippine cities has led to several environmental challenges, which include deteriorating water quality owing to insufficient treatment despite high consumption in urban centers [78], aging infrastructure coupled with ineffective waste management systems [79], and poor waste segregation practices where dry waste (e.g., plastic containers) remains mixed with liquid waste [80]. These conditions create numerous sites of stagnant water, potentially enhancing breeding environments for *Aedes* mosquitoes [81]. Furthermore, unplanned urbanization and substandard housing may also be reinforcing factors for vector breeding [82]. These conditions are evident in highly urbanized cities within the NCR, likely contributing to its consistent status as high-density hotspots (Fig. 1) and high-risk areas for dengue occurrence (Fig. 4). Furthermore, the NCR is the most densely populated region in the country with a population density of 21,765 persons per square kilometer, roughly 60 times that of the national level [46]. This aggravates the condition, as high population density together with microclimatic conditions and rapid mobility predispose major cities to be more affected by dengue, particularly in endemic countries [30].

Socioeconomic variables have greater importance than climatic variables to the ENM model. This is concurrent to previous ecologic niche modeling studies [67, 71]. Factors reflecting urbanization and anthropogenic activity, including nighttime lights, urban-type land cover, and population density, were identified by the model [83]. This may be attributed to the influence of these factors on the distribution of mosquito vectors. Previous studies on the species distribution model for *Aedes aegypti* and other *Aedes* demonstrated that city and construction land as well as distance to built-up surfaces or buildings were important factors influencing vector suitability [62, 63, 84]. In Tehran province, the increase of mosquito densities in urban and peri-urban areas was linked to the doubling rise of dengue cases [85]. These findings suggest that urban environments shape vector suitability conditions and, consequently, drive dengue transmission. A study suggests that the high density of dengue cases in urban areas may be due to increasing temperature [69]. However, this is not evident in our study. Nevertheless, in future projections, high dengue risk may be inevitable owing to rapid socioeconomic developments and global warming [71]. Although population density contributes relatively low to the model, it is a biologically significant factor as it may facilitate dengue transmission [86]. High population density is one of the most important variables in the distribution of *Aedes aegypti* and *Aedes albopictus,* and high-risk areas in Panama [60], Indonesia [70], and Ecuador [16].

The northern Luzon, encompassing Regions I, II, and Cordillera Administrative Region, however, is characterized by mountainous terrains that have been found to have high vector densities [87], a plausible reason for its elevated incidence (Fig. 2). In addition, heavy rainfall and high relative humidity are evident in the region, which are also correlated with high dengue incidence [88]. While dengue control in mountainous areas presents unique challenges owing to ecological, demographic, and infrastructural factors, these findings imply the need for enhanced household and community interventions. The use of mosquito bed nets, fumigation inside homes, and ensuring reliable piped water supply were proven beneficial to reduce dengue risk at the household level [89]. In remote communities, active participation in vector control is critical, especially in identifying and eliminating breeding sites [89].

The Philippines suffered a nationwide epidemic in 2019, which was also observed in multiple countries, especially in the Western Pacific region [18]. According to a World Health Organization situation report dated 25 July 2019, delayed seasonal rains were linked to this phenomenon. The National Dengue Prevention and Control Program (NDPCP) failed in 2019 owing to the dereliction of stakeholders in taking responsibility for dengue prevention, which was further exacerbated by residents storing water owing to unreliable piped water supply and inefficient waste management [90]. Thus, public health authorities should prioritize the enhancement of water infrastructure and waste management systems in conjunction with strengthened vector control programs to effectively reduce mosquito-borne disease incidence. Implementation of more rigorous waste management policies is essential, alongside community-focused dengue prevention campaigns to foster local engagement and initiatives.

The study employed KDE to determine the probability density of dengue cases and IDW interpolation for spatial prediction of incidence rates. KDE can be performed with limited data, such as having only the number and/or exact location of cases. It has also been proven valuable in monitoring vector-borne disease outbreaks in a timely manner and at multiple scales, facilitating the appropriate allocation of resources [91]. IDW, however, requires population data; nevertheless, it is more straightforward to perform. Both KDE and IDW are beneficial analyses depending on the objective of researchers, authorities, and other stakeholders.

In spatial analysis, modeling is performed to describe spatial patterns of disease risk, explain spatial variability, and/or to predict geographical areas suitable for the disease [40, 92]. KDE and IDW were useful in describing the spatial variation of dengue incidence in the Philippines. Furthermore, to better predict dengue risk and identify suitable areas while accounting for exposure variables such as environmental factors, we developed an ecological niche model. The model generated a comprehensive risk map that effectively delineated highly suitable areas and identified the key determinants responsible for the spatial variability in dengue occurrence suitability. Combining these techniques ultimately provides a comprehensive analysis for understanding disease transmission and distribution.

These insights support precise geospatial targeting of interventions and informed virologic surveillance. Strengthening vector control is essential in highly urbanized areas characterized by high nighttime lights, urban land cover, and dense populations. Local governments should prioritize investments in community water infrastructure and waste management, while public health authorities should conduct entomo-virologic surveillance and promote community-based dengue prevention initiatives. Further research is recommended to develop effective strategies for mitigating the impacts of urbanization on vector-borne diseases.

To the best of our knowledge, this is the first application of ecological niche modeling to model the distribution of dengue in the Philippines, which is useful for future risk-based surveillance, genotype monitoring, and control. The study addresses the current gap in knowledge on the highly suitable, high-risk areas for dengue spread and its determinants in the country. The study differs from previous studies that solely relied on statistical techniques and climatic variables, as it utilized maximum entropy model, which is a machine-learning algorithm, and also investigated socioeconomic variables. Furthermore, the study demonstrated the usefulness of KDE and IDW in data-limited scenarios. KDE could be effective for rapid analysis of case data to support immediate response efforts, whereas IDW was more suitable for a comprehensive spatial analysis.

Moreover, the study has demonstrated the great potential of ENM for enhanced virological surveillance and genotype-specific monitoring of dengue in the Philippines. By integrating spatial data of vector occurrences, dengue cases, and genotypic evidence, these models can be useful to identify high-risk areas as well as distribution of certain vector species and dominance of specific DENV genotypes. Entomo-virological surveillance encompassing collecting and genotyping DENV from both mosquitoes and human cases provides real-time data on the circulating serotypes and genotypes, which helps the understanding of current viral dynamics and transmission risk [93, 94]. These endeavors could support early outbreak detection and tailored responses.

This study has certain limitations. The unit of analysis is geographical location rather than individual cases. In addition, the extremely limited data on exact *Aedes* vector locations in the Philippines can lead to less precise local-scale predictions owing to the lack of fine-resolution data. Hence, the study was primarily built on location of provinces and cities where dengue cases had been reported. Modeling using individual level data is not feasible owing to the unavailability of sociodemographic and other individual-level data. Studies at finer spatial scales, such as geocoding case addresses or analyzing barangay-level data, are recommended to provide deeper insights for long-term vector and disease control strategies.

## Conclusions

To our knowledge, this is the first application of ecological niche modeling to predict the distribution of dengue in the Philippines. The comprehensive spatial analysis of combined KDE, IDW, and ENM employed in the study successfully delineated the high-risk, suitable areas for dengue occurrence in the Philippines. Factors relating to urbanization were identified determinants associated with the risk and probable spread of dengue in the country. These results are critical insights for public health authorities to implement risk-based surveillance, genotype-specific monitoring programs, and geospatially targeted control strategies in the Philippines. Infrastructure development in water supply and waste management systems should be prioritized in parallel with entomo-virologic surveillance and community-based dengue prevention initiatives. The integration of methods employed in the study not only provides useful insights but is also transferable to other hyperendemic countries facing similar data limitations.

## Supplementary Information

Supplementary material 1. Additional file 1: Table S1. List of provinces and cities that reported dengue cases. Table S2. Annual optimal bandwidth.

Supplementary material 2. Additional file 2: Fig. S1. Dengue presence locations.

Supplementary material 3. Additional file 3: Fig. S2. Pseudolikelihood bandwidth selection.

Supplementary material 4. Additional file 4: Fig. S3. Contour plots.

Supplementary material 5. Additional file 5: Fig. S4. Correlation matrix of bioclimatic variables.

Supplementary material 6. Additional file 6: Fig. S5. Receiver operating characteristics (ROC) curves for dengue occurrence in the Philippines.

Supplementary material 7. Additional file 7: Fig. S6. Response curves of the most influential predictors for dengue occurrence in the Philippines from 2017 to 2024.

Supplementary material 8. Additional file 8: Fig. S7. Jackknife of test gain of variables in the model.

## Data Availability

Data supporting the main conclusions of this study are included in the manuscript.

## Abbreviations

DALYs: Disability-adjusted life years
DOH: Department of Health
ENM: Ecological niche modeling
GEE: Google Earth Engine
IDW: Inverse distance weighting spatial interpolation
KDE: Kernel density estimation
NDPCP: National Dengue Prevention and Control Program
PIDSR-IS: Philippine Integrated Disease Surveillance and Response Information System
SDG: Sustainable Development Goals
WHO: World Health Organization
