# Supplementary material for: Determination of dengue high-risk areas in the Philippines: a kernel density estimation, inverse distance weighting, and ecological niche modeling
Source: Parasit Vectors. 2025 Dec 23;19:48. doi: 10.1186/s13071-025-07200-4 (PMC12837016; doi:10.1186/s13071-025-07200-4)
Supplement: Supplementary file 1 — Supplementary material 1. Additional file 1: Table S1. List of provinces and cities that reported dengue cases. Table S2. Annual optimal bandwidth. [file 13071_2025_7200_MOESM1_ESM.docx]

**Supplement Tables**

**Table S1**. List of provinces and cities that reported dengue cases.

| **Province/City** | **Longitude** | **Latitude** | **Mean monthly no. of cases*** |
| --- | --- | --- | --- |
| Abra | 17.578321 | 120.803232 | 49.1739 |
| Agusan Del Norte | 9.031258 | 125.535687 | 71.2917 |
| Agusan Del Sur | 8.421598 | 125.728985 | 112.7708 |
| Aklan | 11.609596 | 122.24802 | 154.1758 |
| Albay | 13.209731 | 123.615752 | 29.2717 |
| Angeles City | 15.165213 | 120.549665 | 66.2917 |
| Antique | 11.126782 | 122.068002 | 122.4792 |
| Apayao | 18.105768 | 121.187532 | 92.6947 |
| Aurora | 15.923377 | 121.700327 | 73.2292 |
| Bacolod City | 10.647058 | 122.992972 | 115.2188 |
| Baguio City | 16.399947 | 120.596166 | 183.6947 |
| Basilan | 6.565448 | 122.029089 | 29.2979 |
| Bataan | 14.660473 | 120.454402 | 154.9688 |
| Batanes | 20.552453 | 121.887958 | 30.6271 |
| Batangas | 13.899406 | 121.030006 | 362.3542 |
| Benguet | 16.542684 | 120.698935 | 256.3542 |
| Biliran | 11.596712 | 124.473248 | 29.6237 |
| Bohol | 9.853924 | 124.197679 | 319.5313 |
| Bukidnon | 8.01875 | 125.00693 | 420.1563 |
| Bulacan | 14.978663 | 121.058577 | 706.2813 |
| Butuan City | 8.903163 | 125.577032 | 72.8617 |
| Cagayan | 18.094154 | 121.760607 | 243.9474 |
| Cagayan De Oro City | 8.372778 | 124.602002 | 171.8021 |
| Caloocan City | 14.730091 | 121.029544 | 229.2396 |
| Camarines Norte | 14.143186 | 122.727603 | 29.9063 |
| Camarines Sur | 13.704348 | 123.263146 | 98.5833 |
| Camiguin | 9.171998 | 124.717743 | 30.5682 |
| Capiz | 11.369901 | 122.632934 | 149.1042 |
| Catanduanes | 13.783221 | 124.236447 | 20.6333 |
| Cavite | 14.255164 | 120.86848 | 686.9167 |
| Cebu | 10.355417 | 123.755394 | 553.9375 |
| Cebu City | 10.379238 | 123.859997 | 213.6563 |
| City Of Isabela | 6.650588 | 121.986851 | 48.7396 |
| City Of Las Pinas | 14.441391 | 120.995211 | 81.0729 |
| City Of Makati | 14.553438 | 121.032258 | 100.0625 |
| City Of Malabon | 14.674119 | 120.958556 | 87.4583 |
| City Of Mandaluyong | 14.583087 | 121.041395 | 45.9271 |
| City Of Manila | 14.600622 | 120.98546 | 258.0729 |
| City Of Marikina | 14.647311 | 121.104971 | 69.4271 |
| City Of Muntinlupa | 14.402301 | 121.033601 | 58.7826 |
| City Of Navotas | 14.675139 | 120.935848 | 33.7174 |
| City Of Paranaque | 14.483526 | 121.015008 | 132.1053 |
| City Of Pasig | 14.578867 | 121.085479 | 158.6421 |
| City Of San Juan | 14.602473 | 121.0367 | 25.1935 |
| City Of Santiago | 16.678471 | 121.556776 | 32.2418 |
| City Of Valenzuela | 14.709993 | 120.979164 | 134.8854 |
| Cotabato (North Cotabato) | 7.208721 | 124.870229 | 278.9167 |
| Cotabato City | 7.208623 | 124.231447 | 59.8542 |
| Dagupan City | 16.054136 | 120.34492 | 33.3646 |
| Davao City | 7.26444 | 125.418898 | 320.6875 |
| Davao De Oro | 7.57325 | 126.022926 | 144.0208 |
| Davao Del Norte | 7.58532 | 125.642218 | 204.4271 |
| Davao Del Sur | 7.016216 | 125.345373 | 105.5417 |
| Davao Occidental | 6.098114 | 125.540581 | 36.1011 |
| Davao Oriental | 7.251355 | 126.298228 | 83.2947 |
| Dinagat Islands | 10.170961 | 125.602433 | 35.0482 |
| Eastern Samar | 11.647597 | 125.381085 | 84.9140 |
| General Santos City (Dadiangas) | 6.131293 | 125.130605 | 149.8750 |
| Guimaras | 10.568795 | 122.614091 | 60.4505 |
| Ifugao | 16.848467 | 121.207236 | 99.9792 |
| Iligan City | 8.231113 | 124.372787 | 87.8646 |
| Ilocos Norte | 18.204813 | 120.730124 | 109.9375 |
| Ilocos Sur | 17.247468 | 120.547008 | 155.8021 |
| Iloilo | 11.007873 | 122.605991 | 468.5833 |
| Iloilo City | 10.723489 | 122.555205 | 107.0833 |
| Isabela | 16.987346 | 121.961237 | 295.1458 |
| Kalinga | 17.430975 | 121.279504 | 131.0000 |
| La Union | 16.580543 | 120.424525 | 131.0000 |
| Laguna | 14.241424 | 121.366509 | 600.1563 |
| Lanao Del Norte | 8.026664 | 124.037865 | 161.1875 |
| Lanao Del Sur | 7.796692 | 124.33617 | 178.5604 |
| Lapu-Lapu City (Opon) | 10.286819 | 123.992412 | 109.9681 |
| Leyte | 10.974612 | 124.755849 | 193.1354 |
| Lucena City | 13.936754 | 121.615486 | 38.9634 |
| Maguindanao | 6.869682 | 124.531186 | 77.8961 |
| Maguindanao Del Norte | 7.187669 | 124.243358 | 182.6667 |
| Maguindanao Del Sur | 6.869682 | 124.531186 | 105.7500 |
| Mandaue City | 10.345391 | 123.940865 | 72.8542 |
| Marinduque | 13.391306 | 121.971903 | 32.5169 |
| Masbate | 12.294607 | 123.552328 | 18.6915 |
| Misamis Occidental | 8.324042 | 123.690232 | 172.2292 |
| Misamis Oriental | 8.632576 | 124.791527 | 214.5313 |
| Mountain Province | 17.101296 | 121.129266 | 79.0000 |
| Naga City | 13.643031 | 123.258672 | 32.8737 |
| Negros Occidental | 10.311015 | 122.987131 | 415.7604 |
| Negros Oriental | 9.606913 | 123.033506 | 259.7500 |
| Northern Samar | 12.411099 | 124.791727 | 76.0638 |
| Nueva Ecija | 15.619789 | 121.021668 | 499.5938 |
| Nueva Vizcaya | 16.305431 | 121.171793 | 234.0833 |
| Occidental Mindoro | 12.971959 | 120.892643 | 130.0521 |
| Olongapo City | 14.826789 | 120.306473 | 25.5161 |
| Oriental Mindoro | 12.966297 | 121.263255 | 164.1667 |
| Ormoc City | 11.054199 | 124.65386 | 43.5053 |
| Palawan | 9.978878 | 118.744591 | 256.5938 |
| Pampanga | 15.058512 | 120.647234 | 499.7917 |
| Pangasinan | 15.999504 | 120.312592 | 477.7292 |
| Pasay City | 14.531409 | 121.004 | 54.0105 |
| Pateros | 14.545097 | 121.069479 | 20.6552 |
| Puerto Princesa City | 9.914284 | 118.780386 | 100.8750 |
| Quezon | 14.166484 | 121.962079 | 313.3542 |
| Quezon City | 14.680499 | 121.060474 | 566.9271 |
| Quirino | 16.294548 | 121.595533 | 82.6771 |
| Rizal | 14.617394 | 121.262527 | 555.5417 |
| Romblon | 12.435673 | 122.234517 | 51.5417 |
| Samar (Western Samar) | 11.846482 | 124.940333 | 153.6947 |
| Sarangani | 6.059663 | 125.158334 | 140.3750 |
| Siquijor | 9.185205 | 123.588662 | 34.4409 |
| Sorsogon | 12.854327 | 123.928562 | 42.9889 |
| South Cotabato | 6.284606 | 124.85313 | 279.2813 |
| Southern Leyte | 10.291233 | 125.05195 | 57.5000 |
| Sultan Kudarat | 6.534981 | 124.431281 | 105.3474 |
| Sulu | 5.953179 | 121.054313 | 19.2283 |
| Surigao Del Norte | 9.670729 | 125.737556 | 151.3958 |
| Surigao Del Sur | 8.776365 | 126.113003 | 159.0625 |
| Tacloban City | 11.271349 | 124.955357 | 63.1667 |
| Taguig City | 14.516636 | 121.062346 | 162.9895 |
| Tarlac | 15.478348 | 120.476206 | 449.5000 |
| Tawi-Tawi | 5.239282 | 119.9005 | 14.3111 |
| Zambales | 15.286465 | 120.143828 | 130.9474 |
| Zamboanga City | 7.15592 | 122.145241 | 213.6458 |
| Zamboanga Del Norte | 8.049204 | 122.807342 | 221.3750 |
| Zamboanga Del Sur | 7.679154 | 122.994188 | 219.6146 |
| Zamboanga Sibugay | 7.694735 | 122.725431 | 148.6563 |

**Table S2**. Annual Optimal Bandwidth.

| **Year** | **Optimal Bandwidth** | **No. of Points** | **Maximum Density** | **Mean Density** |
| --- | --- | --- | --- | --- |
| 2017 | 0.4 | 121 | 43863.6575 | 839.088837 |
| 2018 | 0.5 | 121 | 37422.6275 | 1378.45672 |
| 2019 | 0.4 | 121 | 86544.6535 | 2410.33529 |
| 2020 | 0.5 | 121 | 12372.7045 | 489.869355 |
| 2021 | 0.4 | 121 | 20785.77 | 446.22142 |
| 2022 | 0.4 | 121 | 61741.199 | 1458.49044 |
| 2023 | 0.4 | 121 | 41617.3996 | 1198.17288 |
| 2024 | 0.4 | 122 | 77203.6651 | 2285.37481 |
