# Supplementary figures and images for: Determination of dengue high-risk areas in the Philippines: a kernel density estimation, inverse distance weighting, and ecological niche modeling
Source: Parasit Vectors. 2025 Dec 23;19:48. doi: 10.1186/s13071-025-07200-4 (PMC12837016; doi:10.1186/s13071-025-07200-4)

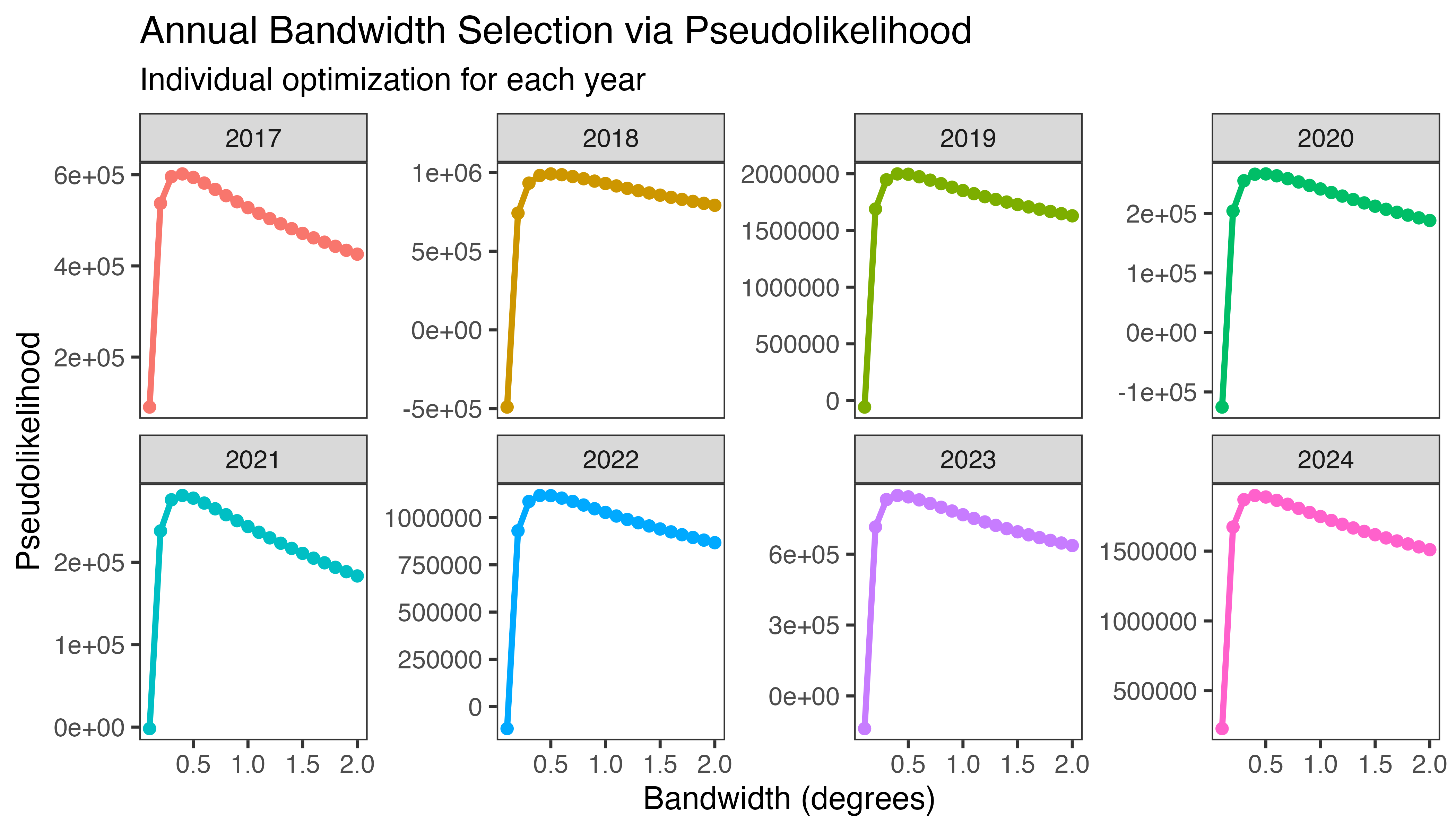

Supplement: Supplementary file 3 — Supplementary material 3. Additional file 3: Fig. S2. Pseudolikelihood bandwidth selection. [file 13071_2025_7200_MOESM3_ESM.png]

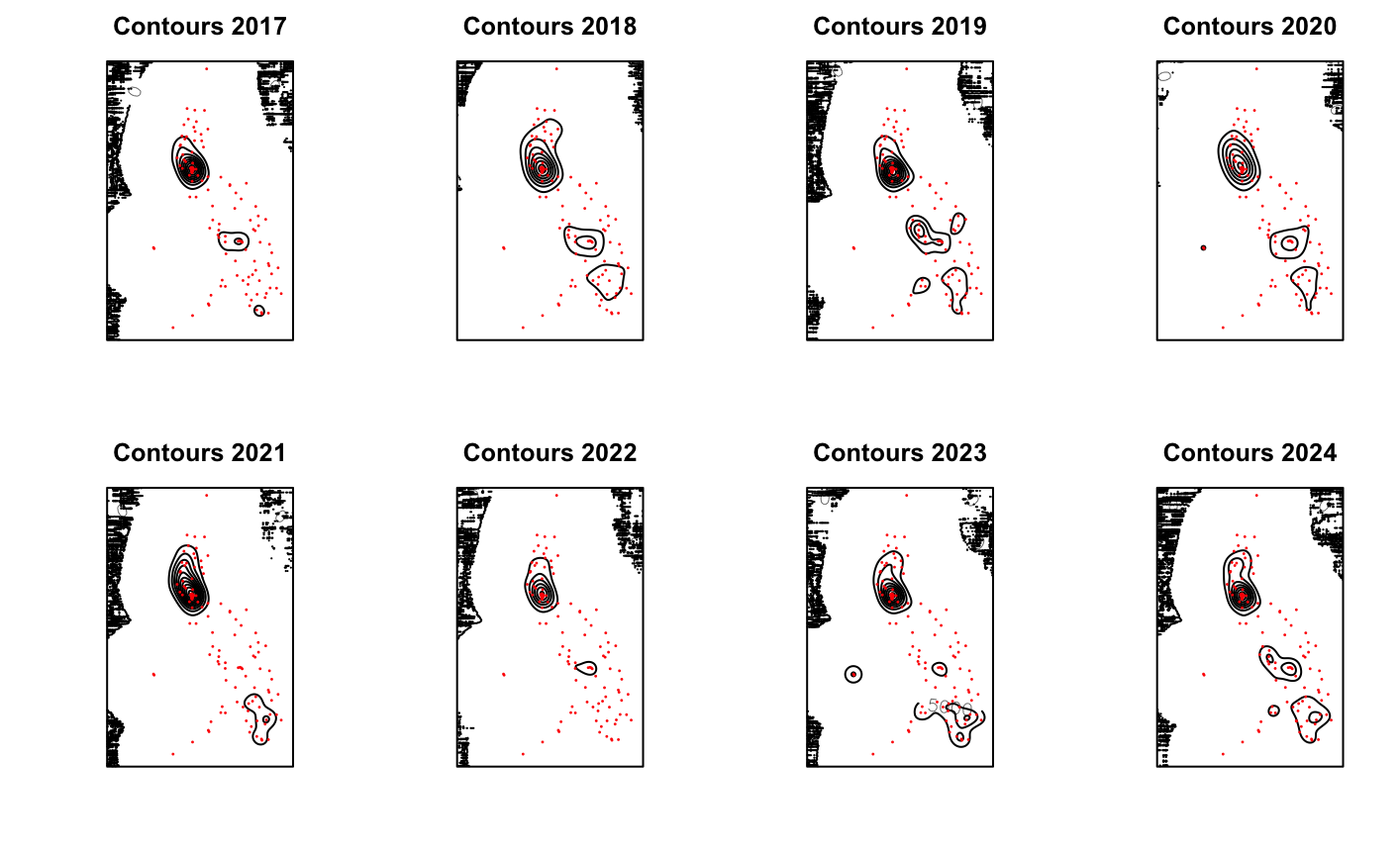

Supplement: Supplementary file 4 — Supplementary material 4. Additional file 4: Fig. S3. Contour plots. [file 13071_2025_7200_MOESM4_ESM.png]

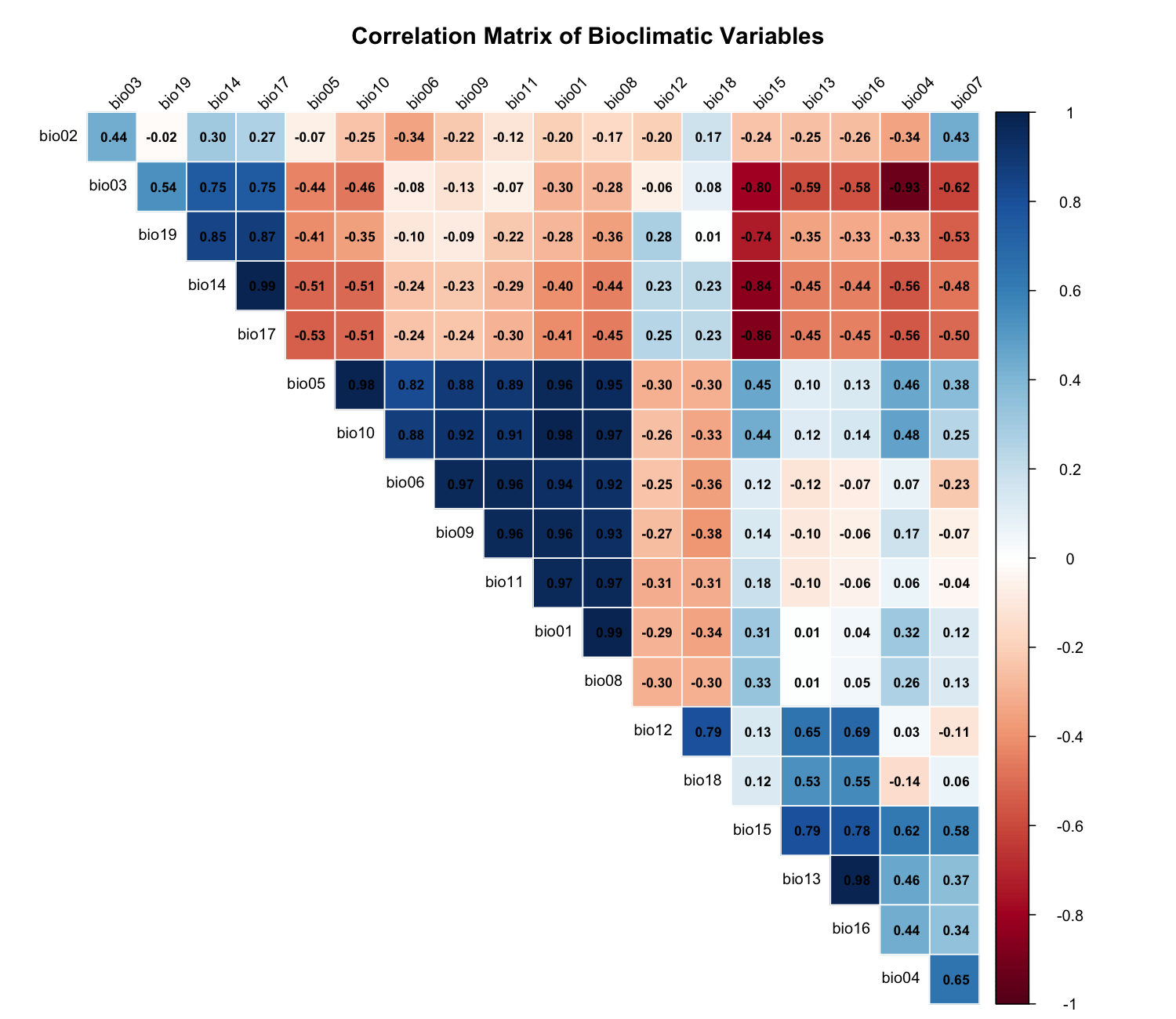

Supplement: Supplementary file 5 — Supplementary material 5. Additional file 5: Fig. S4. Correlation matrix of bioclimatic variables. [file 13071_2025_7200_MOESM5_ESM.png]

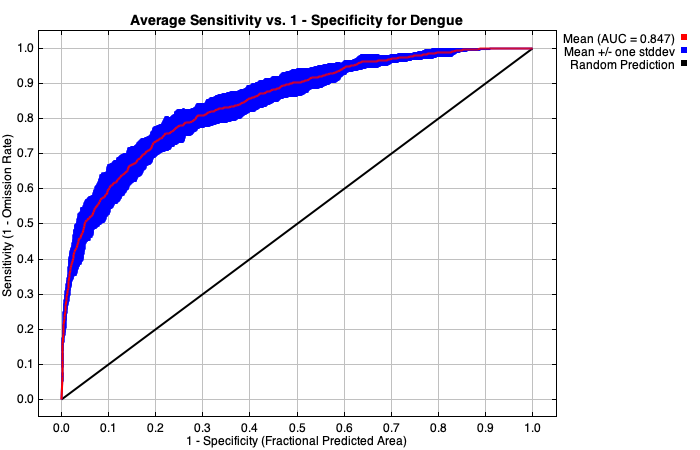

Supplement: Supplementary file 6 — Supplementary material 6. Additional file 6: Fig. S5. Receiver operating characteristics (ROC) curves for dengue occurrence in the Philippines. [file 13071_2025_7200_MOESM6_ESM.png]

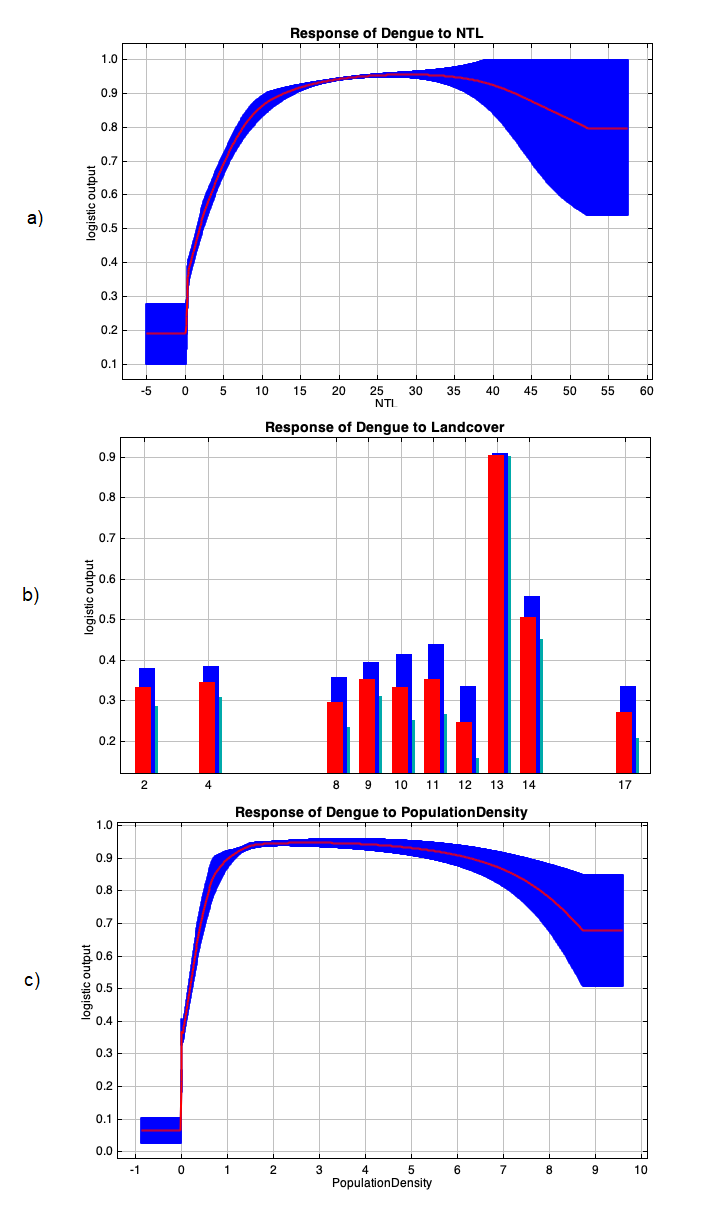

Supplement: Supplementary file 7 — Supplementary material 7. Additional file 7: Fig. S6. Response curves of the most influential predictors for dengue occurrence in the Philippines from 2017 to 2024. [file 13071_2025_7200_MOESM7_ESM.png]

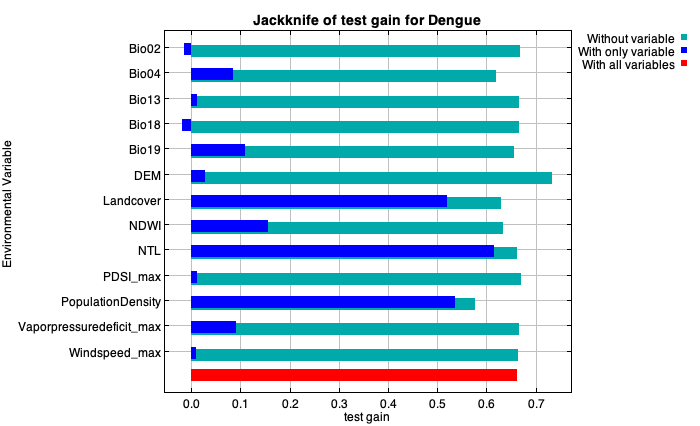

Supplement: Supplementary file 8 — Supplementary material 8. Additional file 8: Fig. S7. Jackknife of test gain of variables in the model. [file 13071_2025_7200_MOESM8_ESM.png]
